# Enhancing Weathering Resistance of Wood—A Review

**DOI:** 10.3390/polym13121980

**Published:** 2021-06-17

**Authors:** Vlatka Jirouš-Rajković, Josip Miklečić

**Affiliations:** Department of Wood Technology, Faculty of Forestry and Wood Technology, University of Zagreb, Svetosimunska 23, 10000 Zagreb, Croatia; vjirous@sumfak.unizg.hr

**Keywords:** wood surface, weathering, photostabilizers, coatings, nanoparticles, plasma deposition, chemical modification, thermal modification

## Abstract

Wood is a truly sustainable and aesthetically pleasant material used in indoor and outdoor applications. Every material, including wood, is expected to have long-term durability and to retain its original appearance over time. One of the major disadvantages of wood is the deterioration of its surface when exposed outdoors, known as weathering. Although weathering is primarily a surface phenomenon, it is an important issue for wood products as it affects their appearance, service life, and wood-coating performance. To encourage the use of wood as a material for joinery and other building components, the results of research into increasing the weathering resistance of wood are extremely significant. The development of weathering protection methods is of great importance to reduce the maintenance requirements for wood exposed outdoors and can have a major environmental impact. There are various methods of protecting wood surfaces against weathering. This paper provides a literature survey on the recent research results in protecting wood from weathering. The topics covered include surface treatments of wood with photostabilizers; protection with coatings; the deposition of thin film onto wood surfaces; treatments of wood with inorganic metal compounds and bio-based water repellents; the chemical modification of wood; the modification of wood and wood surfaces with thermosetting resins, furfuryl alcohol, and DMDHEU; and the thermal modification of wood.

## 1. Introduction

Wood is a renewable, natural material that has been used for centuries in various applications, mostly for furniture and in building construction. In addition to many desirable properties, such as an attractive appearance, good strength, low density, and good insulating properties, wood also has some less desirable properties, such as hygroscopicity, flammability, susceptibility to biological attack, and surface degradation due to weathering.

Weathering can be defined as the slow degradation of the wood surface that occurs through the combined effect of sunlight, water, oxygen, temperature, and atmospheric pollution [1]. Colour change is the first sign of complex chemical reactions on the surface of wood exposed to weather, initiated by solar radiation, especially the ultraviolet (UV) part of the spectrum. UV radiation with its high amount of energy can cause the breakdown of bonds in the polymeric molecules of wood and photochemical reactions that lead to the depolymerization of lignin and cellulosic polymers in the wood cell wall [2,3]. Of all wood polymers, lignin is the best absorber of UV light with chromophore functional groups capable of absorbing a broad spectrum of UV light in range of 250–400 nm [4]. Lignin absorbs more UV radiation than cellulose, resulting in more photochemical degradation reactions that involve free radicals [5]. The phenolic hydroxyl groups in lignin react with UV radiation to form aromatic free radicals (phenoxy radicals), which further react with oxygen to form quinoid structures that are responsible for the yellowing of wood [6,7]. The surface of light coloured woods usually turns yellow or brown, and darker types of wood that are rich in extractives may first fade and then turn yellow or brown. However, after a long period of natural exposure (depending on climatic conditions) the surfaces of all species turn grey because the photodegraded lignin portions are leached from the wood surface, and a cellulose-rich surface layer remains on top [5]. The growth of fungi and moulds [3] and the dust particles which penetrate the porous structure of wood may also contribute to the grey colour [8]. In addition to colour change, the changes in surface texture are the most visible consequence of wood weathering. The erosion of the wood surface is the result of chemical degradation of the wood by UV and visible light followed by a mechanical abrasion due to rain and wind [9]. Water leaches out products from photodegradation and hydrolysis and washes away loosened cellulose fibres, causing a rough surface and weight loss [3,10]. Extractives are also leached from the wood surface during weathering, and wood becomes less water repellent [10]. Furthermore, stresses caused by the wetting and drying of the wood result in the formation of surface checks that may grow into large cracks [5,11,12]. The rate of erosion depends on wood density [9] and, in general, is 3 mm per century for hardwoods and 6 mm per century for softwoods [10]. The effect of the photodegradation is limited to a thin surface layer of the wood due to the limited penetration of wood by light. The depth of sunlight penetration into the wood is found to be dependent on the wavelength of the light [13]. Although earlier research by Hon and Ifju [14] indicated that UV light penetrates wood 75 µm, more recent research reported that UV light penetrates the wood deeper, mostly up to 150 µm [15,16,17,18,19]. It has also been shown that visible light up to the violet region contributes to surface discolouration [17,18]. In addition to these visible changes in the wood surface, weathering affects the wettability of the wood surface [10,20], the surface layer strength [21,22,23], chemical composition [6,8,24,25], and microscopic structure of the wood surface [26,27,28,29].

The mechanism of weathering as well as its influence on the surface properties of wood has been investigated by many authors. A good review of the research in this area is given in several review papers [1,3,5,10,12,30,31]. Although weathering is primarily a surface phenomenon, it is an important issue for wood products exposed outdoors as it affects their appearance, service life, and wood-coating performance.

The weathering durability of wood can be tested using outdoor exposure or using special devices that can simulate outdoor weathering. The natural weathering is performed in outdoor fields with real sunlight but without an exposure parameters control. The device for artificial weathering is usually equipped with temperature control, a light irradiation source (xenon lamps or fluorescent UV lamps), water spray, and condensation units. The results of natural exposure are difficult to compare with the results of accelerated exposure due to different mechanisms of material degradation. In addition, biological impact and air pollution are lacking in artificial weathering. Moreover, differences in weathering behaviour exist among various species, and these differences are pronounced at the beginning of the weathering and decrease with the extension of the weathering [32].

In order to improve the weathering durability of the wood and achieve a long life for wood products, the various methods of protecting wood against weathering are applied. The aim of this paper is to review recent developments in the methods of protecting wood from weathering. The paper is divided into the following chapters: Section 2. Surface treatments with photostabilizers; Section 3. Protection by coatings; Section 4. Thin film deposition onto wood surfaces; Section 5. Treatments of wood with inorganic metal compounds and bio-based water repellents; Section 6. Chemical modification of wood; Section 7. Modification of wood and wood surface with thermosetting resins, furfuryl alcohol, and DMDHEU; and Section 8. Thermal modification.

## 2. Surface Treatments with Photostabilizers

Photostabilizers are additives used to prevent the photodegradation of wood and improve the performance of transparent coating. The most common photostabilizers used to protect the wood from photodegradation and improve the performance of transparent coatings are UV absorbers (UVA) and radical scavengers. The most common organic UV absorbers for coatings are2-(2-hydroxyphenyl)-benzotriazole (BTZ) and 2-hydroxyphenyl-s-triazine (HPT) derivatives [33]. Due to their chemical structure, they absorb energy from UV radiation and dissipate it as heat through a reversible chemical rearrangement. There are also inorganic UV light stabilizers, the so-called mineral screeners which are ideally nanoparticulate materials comprised of titanium dioxide (TiO_2_), zinc oxide (ZnO), or cerium oxide (CeO_2_) [33]. They absorb and reflect the UV and VIS spectra of radiation [34]. Radical scavengers, also called primary antioxidants, react with the propagating radicals, such as peroxy, alkoxy, and hydroxyl radicals, making them inactive [35]. The most used commercial antioxidants are hindered phenols and hindered amine light stabilizers (HALS). For optimal wood protection against photodegradation, UVA are usually combined with HALS and exhibit a synergistic effect.

It has been shown that pretreatment of the wood surface prior to the application of a clear topcoat can effectively protect lignin from photodegradation. For improved colour stability of lighter wood species in the interior and for better performance of clear and transparent pigmented wood coatings in outdoors applications, the so-called lignin stabilization concept has been developed. The newly developed HALS is applied directly on wood as a dilute aqueous solution or in a primer formulation, followed by the application of subsequently coating layers containing the UV absorber based on HPT chemistry [36]. The special monomeric HALS [37] acts as an effective lignin stabilizer, trapping the radicals at the wood surface formed by visible light not screened by UVA (>400 nm). Several studies have been carried out to exam the efficacy of different surface treatments in reducing the discolouration of wood surfaces induced by light using HALS [36,38,39], nanoparticles [40,41], or combinations thereof [42,43]. Yang et al. [39] reported that pretreatment of the wood with lignin stabilizer before applying a clear top coating contributed to the better protection of the Southern pine wood surface from photodegradation. The best protection was achieved by pretreatment of the wood surface with a lignin stabilizer followed by waterborne clear coating with an added blend of UV absorber and HALS. Forsthuber and Grüll [42] found that monomeric HALS for lignin stabilization in an aqueous primer in combination with transparent topcoats comprising organic UVA and inorganic TiO_2_ screeners significantly improved the colour retention of Norway Spruce (*Picea abies* (L.) H.Karst.) wood.

Pánek et al. [43] examined the ability of different UV-stabilizing penetrating treatments containing UV stabilizers, HALS, nanoparticles of TiO_2_ and ZnO, and combinations thereof to reduce the discolouration of four types of wood species (oak, larch, Douglas fir, and spruce) during accelerated exposure to UV light. They found that the effectiveness of each treatment in the photo stabilization of the wood surface depended on the type of wood and that a synergistic effect was achieved when the active substances were used together compared to when they were used individually. Clausen et al. [40] reported that after 12 months of outdoor exposure, Southern pine wood samples vacuum treated with nano-ZnO exhibited reduced greying, moderate checking, and substantial resistance to water absorption (at a concentration of 2.5% or greater) compared to untreated and unweathered wood samples.

Chang et al. [44] examined the colour changes of Taiwania (*Taiwania cryptomeriodes* Hayata) wood treated with UV absorbers (Tinuvin-1130 and Tinuvin-292), PEG (polyethylene glycol), or SCB (semicarbazide) followed by polyurethane coating containing Tinuvin-1130 during artificial exposure to UV light. They reported that pretreatment with a combination of UV absorbers or PEG followed by polyurethane coating containing Tinuvin-1130 photostabilizer reduced wood discolouration by 30% compared to wood without pretreatment.

### 2.1. Treatments with Natural Photostabilizers

Since some phenolic wood extractives have been shown to have better antioxidant capacities than synthetic antioxidants, an innovative approach might be to protect the wood with natural extractives coming from durable wood species [7,45,46,47]. Impregnation of pine and poplar wood with an extract of more photoresistant wood species was shown to provide a more stable colour [45]. However, the impregnation of beech wood with natural extracts of mimosa (*Acacia mollissima*) and quebracho (*Schinopsis lorentzii* (Griseb.) Engl.) followed by coating with clear lacquer was found to be ineffective in reducing colour changes during accelerated exposure to UV light [48]. The heartwood extracts of *Acacia confusa* Merr.were found to be effective at protecting wood from photodegradation via UV light absorption and free radical scavenging [49,50,51]. Their ability to prevent lignin degradation has been shown to be comparable to the commercially available benzotriazole-type UV absorber and hindered amine light stabilizer [52]. It has been shown that condensed tannins in *Acacia confusa* Merr. heartwood can reduce lignin photodegradation due to their singlet oxygen quenching abilities and phenoxyl radical scavenging abilities [53,54]. The most abundant flavonoids in *Acacia confusa,* melanoxetin and okanin, were shown to have a good UV absorptivity, singlet oxygen quenching ability, and phenoxyl radical scavenging ability [55]. These natural photostabilizers are shown to be a good alternative to synthetic photostabilizers due to their low toxicity and biocompatibility and will certainly be the subject of future research.

### 2.2. Grafting of Photostabilizers

In order to improve the effectiveness of UV absorbers in preventing the photodegradation of wood, several authors have investigated the chemical bonding of the UV absorbers to wood for protecting wood from photodegradation. Benzophenone or triazine UV absorbers containing an epoxy group can be enduringly bonded to wood at high temperatures in the presence of an amine catalyst [56]. Williams [57] first reported grafting of 2-hydroxy-4-(2,3-epoxypropoxy)-benzophenone (HEBP) to western redcedar (*Thuja plicata* Donn ex D.Don) and the performance of the HEBP-grafted wood during artificial accelerated weathering. The UV stabilizer HEBP contains a UV stabilizing group and a glycidyl ether (epoxide) that can react with wood hydroxyls [57]. The chemically bound system reduced the erosion of uncoated wood, prolonged the life of polyurethane coating, and improved wood colour retention both with and without a clear coating. Kiguchi and Evans [58] studied the potential of epoxy-functionalized UV absorbers grafting as a photoprotective treatment for three wood species (*Pinus sylvestris* L., *Cryptomeria japonica* D. Don and *Populus* spp.) and the reaction conditions necessary for grafting HEPBP to wood. They found that in the presence of amine catalyst at temperatures in excess of 80 °C, HEPBP was grafted to wood, resulting in permanent weight gains. Grafting of HEPBP was more effective than chromium trioxide in preventing the degradation of cellulose during natural weathering and as effective as chromium trioxide in the protection of lignin. They also reported that undesirable colour changes arising from modification were smaller for grafting compared to the changes caused by chromium trioxide. Photochemical changes during weathering were also reduced on grafted wood, and the performance of transparent coatings was improved. However, the grafting of epoxy-functionalized UV absorbers improved the performance of one tested coating applied to the grafted wood veneer, while the other tested clear coating did not show an improvement in performance during outdoor natural weathering compared to untreated wood veneer [13]. The grafting of epoxy-functionalized triazine type UV absorbers has been also shown to protect wood surfaces against UV radiation. Triazine-grafted veneers showed a greater colour change than HEPBP-grafted veneers during accelerated weathering, but the mass losses were similar [13].

Grelier et al. [59] synthesized different UV stabilizers bearing an isocyanate function. They were grafted to medium density fibre (MDF) board and to fir (*Abies grandis* (Douglas ex D.Don) Lindl.) and European oak woods using microwave activation, and the photoinduced discolouration was determined after artificial weathering. The grafting of UV absorbers to the wood surface has been shown to allow a higher efficiency of protection when compared to the action of the additives adsorbed on the material surface. The best protection was found when the UV absorber was used in conjunction with polyethylene glycol or hindered amine light stabilizers and when grafted onto the wooden materials.

The treatment of maritime pine wood (*Pinus pinaster* Aiton) with a copper-amine solution followed by the grafting of a mixture of polyethylene glycol and hydroxyphenilbenzo-triazole using an urethane bond from isocyanate was shown to be effective against wood surface discolouration [60]. The microwave grafting of UV phenolic absorber onto copper-amine treated maple wood (*Acer rubrum* L.) was found to be efficient in reducing colour change and surface roughness during accelerated weathering [61].

Grelier et al. [62] grafted polystyrene-maleic anhydride copolymer containing polyethylene glycol chains and benzotriazole UV absorber (with acyl azide functionality) to pine (*Pinus sylvestris* L.) wood. This treatment improved the colour stability of wood against UV light and the adhesion of clear coatings on grafted wood exposed to artificial accelerated weathering. However, the coated wood did not have the expected colour stability after accelerated weathering in the presence of water possibly due to the sensitivity of the coatings.

Evans and Chowdhury [63] synthesized polymeric UV absorbers from epoxy-functionalized UV absorber 2-hydroxy-4(2,3-epoxypropoxy)-benzophenone (HEPBP) and dicarboxilic acid anhydrides (maleic, phtalic, and succinic anhydride) and examined the ability of the UV absorbers to photostabilize veneers of yellow cedar (*Chamaecyparis nootkatensis* (D.Don) Spach.) during accelerated weathering. It has been shown that HEPBP reacted with phthalic anhydride to create a polyester-type UV absorber which is an effective photoprotective treatment for wood. HEPBP-phtalic anhydride was more effective at restricting the weight and tensile strength losses of wood veneers after accelerated weathering than treatments with UV absorbers formed from HEPBP and maleic or succinic anhydride. This absorber showed a strong absorption of UV radiation with an absorption peak that coincides with that of lignin and formed a leach-resistant film at wood surfaces. The results of this study show that the copolymerization of functionalized UV absorbers with other compounds to increase the molecular weight of the UV absorber can be a successful strategy in protecting wood from photodegradation. By changing the reactive UV absorbers and copolymers or by increasing the weight gains of veneers, the efficiency of the polymer UV absorbers can be increased. Olsson et al. [64] studied the photostabilizing effect of the grafting of 2-hydroxy-4(2,3-epoxypropoxy)-benzophenone (HEPBP) to Scots pine wood veneers alone or in combination with epoxy-functionalized soybean oil. The combination of epoxy-functionalized UV absorber and commercially available epoxy-functionalized soybean oil reduced the colour changes of wood surfaces during accelerated weathering, and the treated veneers exhibited reduced brittleness compared to untreated samples or samples treated with only HEPBP. However, the overall effect of using the combination of HEPBP and epoxy-functionalized soybean oil was lower than expected, but this interesting approach to combining reactive absorbers and hydrophobes that may synergistically protect the wood surface from photodegradation might inspire new similar research. However, the cost of grafting photostabilizers to a wood surface is quite high, and the process is unsuitable for DIY market. The grafting of UV stabilizers could be used as an industrial treatment, but further research is needed to reduce the cost of the method.

## 3. Protection with Coatings

The common way to protect wood from weathering is to protect it with a wide range of coatings, such as paints, varnishes, stains, and water repellents [65]. The primary function of a coating is to protect the wood from the two main causes of the natural weathering process, UV radiation and moisture, and to help maintain its appearance. Most studies and patents related to the protection and preservation of wood are focused on coating modification [66]. Coating’s performance is considered through a change of colour and gloss, loss of adhesion brittleness, chalking, peeling and blistering, and structural changes in the coating [67]. In selecting the proper coating, the main problem is to determine the balance among preserving the natural appearance of wood, protecting the wood surface, and increasingly stringent environmental regulations [68]. Environmental conditions negatively affect the physical and mechanical properties and chemical composition of the coating by creating new functional groups or the fragmentation of cross-linked macromolecules [69]. Furthermore, the absorption of UV radiation into the coating and the wood under the coating can cause serious and complex chemical reactions that result in the loss of the protective function of the coating and in the deterioration of the coating and wood substrate [70,71]. In order to extend the lifetime of wood and maintain the natural and attractive appearance of wood, research and development of clear coatings with minimal use of harmful chemicals has become very important for wood finishing [67]. However, clear coatings transmit harmful solar radiation which causes changes on the wood surface [5,71,72,73]. Most clear coatings fail after two years of outdoor exposure in temperate climates and fail after one year in tropical climates [74].

There are three methods for the protection of a wood coating system from harmful solar irradiation [71]. The first is based on reflection (filter effect) using pigments, the second on the suppression of the reaction by removing newly formed free radicals using HALS, and the third on the absorption of UV radiation before the formation of free radicals using UV absorbers. Pigments are insoluble fine-size materials used in coatings to improve their performance [75]. Although pigments are best known for their interaction with visible light and hence colour, they also absorb or scatter other parts of sun radiation [76]. The main disadvantage of adding pigments into the coating is completely hiding the texture and colour of the wood (opaque coatings) or changing the colour of the wood without hiding wood texture (stains). HALS can be applied directly on wood as an aqueous solution or primer, and they can also be used as an additive in a topcoat [77]. According to the research of [7], HALS can also be an effective protection against the loss of gloss, microcracking of clear coatings, and surface erosion. Saha et al. [78] state that lignin stabilizers play a significant role in protecting wood from UV light.

Organic UV absorbers that are added into the coating to protect wood and coatings from harmful UV radiation are based on benzophenone, benzotriazole, triazine, malonate, and oxalanilide [67]. Benzotriazole is the most important organic UV absorber for many clear coatings due to its high absorption of UV light and low absorption of visible light [79]. However, organic UV absorbers, due to their relatively low molecular weight, can migrate to the surface of the coating or into the wood. They are also susceptible to degradation due to their organic nature [80]. Allen et al. [81] found that the addition of organic UV absorbers into the coating protected the wood from discolouration at the beginning of artificial weathering, but this protection was not long lasting due to the migration and decay of the absorbers during artificial weathering. In coatings for outdoor use, organic UV absorbers are added in a concentration of 1–5%, and in that concentration, they reduce the coating transparency [82]. There are various data on the effectiveness of benzophenone in wood protection from weathering effects. Rao et al. [79] found that coating with benzophenone led to less photodegradation of bamboo than coating with ZnO nanoparticles during accelerated weathering. Furthermore, they found that the chemical structure of the coating becomes more stable to accelerated weathering after the addition of organic UV absorbers with increasing bamboo durability. However, Akbarnezhad et al. [83] found that bezophenone-modified acrylic coating did not reduce the impact of natural weathering on beech wood. The efficiency of UV absorbers is determined by their absorption properties, concentration in the coating, coating thickness, chemical interaction with the binder, and other additives in the coating during photodegradation [69]. Inorganic UV absorbers can protect the coating and wood substrate from UV radiation for a long time because they do not disintegrate and migrate to the surface of the coating during exposure to outdoor environmental conditions; however, to some extent, they change the colour of the coating. Many metal oxides are known to absorb UV radiation, and the most commonly used are TiO_2_, ZnO, CeO_2_, and iron oxides. Forsthuber and Grüll [42] found that TiO_2_ microparticles reduce wood discolouration, but due to their absorption in the visible part of sunlight, they blur the coating, which can be a disadvantage on darker wood species.

**Figure 1:**
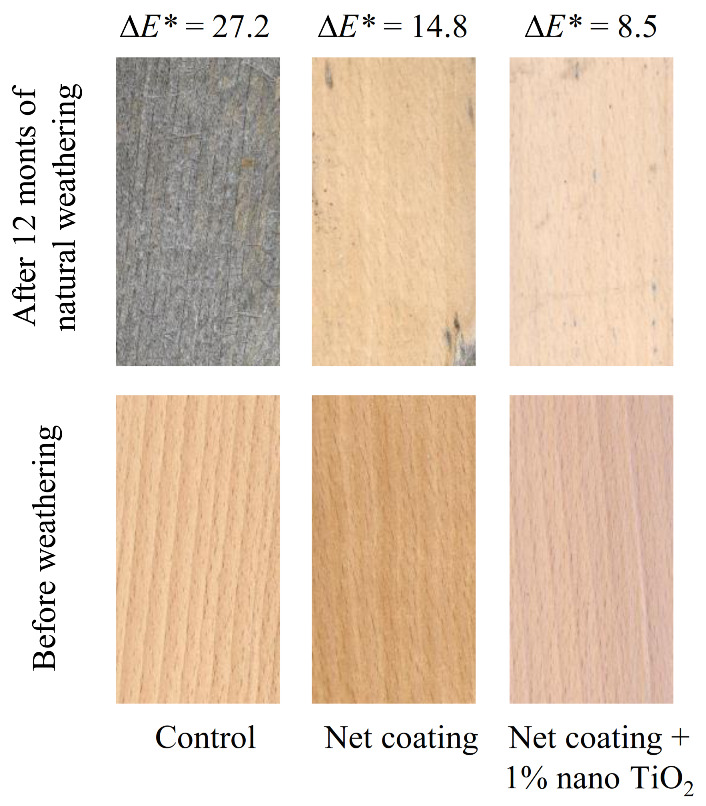
Colour change of uncoated (control) and coated beech wood with net coating and net coating with 1% nano TiO_2_ after 12 months of natural weathering.

In order to increase the durability of coating and wood while maintaining the transparency of coating, inorganic UV absorbers in nano size are increasingly used in addition to HALS and organic UV absorbers [34,84]. The accelerated development of nanotechnology has enabled the application of nanoparticles of metal oxides to protect coatings and wood surface without significantly affecting the transparency of the coating. Nikolic et al. [85] state that inorganic nanoparticles can be efficient UV absorbers in coatings depending on the type, loading size, and dispersion of the nanoparticles. Nanomaterials can be incorporated into the coating by two different methods: mixing or in situ [86]. In the first method, the appropriate nanomaterial is added into the coating, after which a force is applied to obtain an emulsion. Nanoparticles of UV absorbers can be added into the coating as a powder or emulsion. When mixing nanoparticles with a coating, it is important to achieve the most uniform dispersion of nanoparticles in the coating and ensure the compatibility of nanoparticles with the coating ingredients to avoid a decrease of coating transparency, an increase of coating viscosity, a sudden deposition of nanoparticles, and a large agglomeration of nanoparticles in the coating. In the second method of nanomaterials incorporation into the coating, nanomaterials are added directly to the monomers followed by their polymerisation. Cristea et al. [87] found a high compatibility of TiO_2_ and ZnO nanoparticles with an acrylic resin-based coating, and thus an increase in its durability after accelerated weathering. They also state that the aggregation of nanoparticles in the wet and dry coating film can be reduced by mixing the emulsion of nanoparticles instead of the powder of nanoparticles with the coating. Fufa et al. [88] reported that the addition of TiO_2_ nanoparticles into the coating reduced discolouration and chemical changes on the coated wood surface during accelerated exposure to sunlight and water. Miklečić et al. [89] found that TiO_2_ and ZnO nanoparticles increase the colour stability of waterborne polyacrylate coating during natural and accelerated weathering, and the result was better with a higher concentration of nanoparticles. However, ZnO nanoparticles increased the brittleness of the waterborne polyacrylate coating. The protective effect of TiO_2_ nanoparticles on the colour stability of beech wood can be seen in Figure 1.

Moya et al. [90] found that TiO_2_ nanoparticles added into the varnish reduced the colour change of tropical wood species during natural and accelerated weathering. Akbarnezhad et al. [83] found that an acrylate coating with ZnO nanoparticles reduced colour change and mould growth on beech wood during natural weathering. Moreover, studies have also shown that the combined protection of coatings with UV absorbers (organic and inorganic) and HALS has a positive effect on the protection of wood from UV radiation. Thus, a positive effect of a combination of ZnO nanoparticles, benzotriazole, and HALS in a penetrating coating system on the colour stability of oak wood during UV radiation was reported by [91]. Furthermore, Rao et al. [79] found that the combination of benzotriazole and ZnO nanoparticles showed the best performance for colour stabilisation of bamboo due to UV radiation. However, most studies have shown that UV absorbers have a positive effect on the performance of clear coating, but clear coatings can fail on wood due to the influence of water and fungi in combination with solar radiation [74].

Although nanotechnology has more effectively solved various problems in wood science than traditional methods, it is still necessary to conduct a large number of tests and introduce a large number of regulations before the commercial application of nanotechnology in protecting wood and coatings, especially in interior environments [66]. There are considerable concerns growing about the impact of nanomaterials on human health and the environment. Due to their small size, nanomaterials can have a negative effect on the respiratory and digestive tracts, eyes, and skin [92,93].

## 4. Thin Film Deposition onto Wood Surfaces

### 4.1. Direct Deposition of Nanoparticles onto Wood Surfaces

Nanotechnology provides new opportunities to protect wood from UV radiation and weathering. Nanoparticles are mostly added to wood coatings or pretreatments, but direct deposition/growth of nanoparticles on the wood surface is also possible. Yu et al. [94] reported on the formation of ZnO nanofilms on the surface of Chinese fir wood using a two-step process consisting of an immersion treatment of the samples in ZnO nanosol followed by further particle growth on the surface, which was achieved by another immersion treatment. The photoprotection effect of ZnO nanofilms was evaluated during accelerated weathering and it was found that nano ZnO modification significantly improved the photostability of Chinese fir wood.

**Figure 2:**
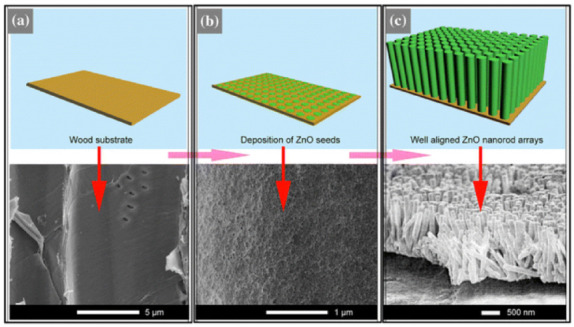
Schematic illustration of the formation process for the ZnO nanorod arrays on the wood surface ((**a**): row wood surface, (**b**): densely deposed ZN crystal seed onto wood surface, (**c**): large-scale ZnO nanorod arrays) [96]. Reprinted with permission from Springer Nature: Springer Nature, *Journal of Materials Science* (Improved UV resistance in wood through thehydrothermal growth of highly ordered ZnOnanorod arrays, Sun, Q. et al.), 2012.

It has been shown that ZnO nanostructures can be also formed on a bamboo surface using a simple two-step process consisting of seed coating in ZnO nanosol and crystal growth in a zinc salt aqueous solution [95]. Using this approach, the bamboo surface can be simultaneously functionalized with photostability, antibacterial, and antifungal activities. Sun et al. [96] grew highly ordered ZnO nanorod arrays on a wood surface using a facile one-pot hydrothermal method (Figure 2) and evaluated the UV resistance of the original wood and the ZnO/wood composite. The ZnO/wood exhibited an improved UV resistance compared to original wood probably due to the excellent UV absorption of the well-aligned ZnO nanorod arrays.

Fu et al. [97] fabricated well-aligned ZnO nanorods on poplar (*Populus tomentosa* Carr.) wood surfaces by the conventional hydrothermal method and by the microwave-assisted hydrothermal method and compared the crystallographic data, the microstructure of the nanorod layers, and their ability to protect the wood surface against UV radiation. The reaction time of the microwave-assisted hydrothermal method was significantly shortened compared to the conventional hydrothermal method. ZnO nanorods produced by the microwave-assisted hydrothermal method showed better crystallinity, smaller diameters, and narrower size distributions than ZnO nanorods produced by the conventional hydrothermal method. The UV-protecting effects of the ZnO nanorod layer produced by the microwave-assisted hydrothermal method was the same as the UV-protecting effect of the ZnO nanorod layer produced by the conventional hydrothermal method.

Guo et al. [98] used a chemical bath deposition process for the deposition of ZnO nanostructures on a Spruce wood (*Picea abies* (L.) H.Karst.) surface. The morphology of ZnO nanostructures was adjusted during the growth process by ammonium citrate, resulting in platelet structures, and by aluminium nitrate, resulting in nanorod arrays. The performance of wood modified with these two nanostructured coatings was assessed during accelerated weathering and during UV irradiation. It has been established that these two coatings had almost equal performance in terms of UV protection, but in the presence of water spray, the dense ZnO coating with a platelet structure protected the wood much better against weathering than the ZnO nanorod array coating. The ZnO nanorod coating exhibited strong colour changes and crack formation on the wood samples during accelerated weathering, indicating a limitation of using ZnO nanorod coated wood for outdoor applications. 

TiO_2_ nanoparticles were reported to be successfully deposited on an Iranian beech (*Fagus orientalis* Lipsky) wood surface by the sol-gel deposition process [99]. The TiO_2_-coated wood samples exhibited a protective behaviour against UV light and water.

Submicrospheres of anatase and rutile TiO_2_ have been in situ deposited on wood surface using the one-pot hydrothermal method, and the UV resistance of TiO_2_-modified wood samples was assessed during accelerated weathering [100]. The resistance to the UV radiation of rutile TiO_2_-modified wood samples proved to be better after accelerated weathering than the UV resistance of anatase TiO_2_-modified wood samples. This is probably due to the low photocatalytic oxidation capability of rutile TiO_2_, its strong UV absorption, and its high light scattering capability. However, Hernandez et al. [101] reported that the photocatalytic activity of TiO_2_ may be crucial for the performance of nanoparticles in the protection against UV radiation on wood surfaces.

Wang et al. [102] wanted to develop a hydrophobic nanocoating on the surface of Chines fir wood (*Cunninghamia lanceolata* (Lamb.) Hook.) which, in addition to protection against UV radiation, also provides water repellence. They used a two-step treatment by first growing TiO_2_ on the wood substrate using a sol-gel process, followed by silylation. The formed TiO_2_ coatings were transparent and showed a strong absorption of UV radiation, thus giving the wood substrate increased photostability depending on the concentration of TiO_2_. However, such two-step processes are probably too expensive for wide commercial application. A surface of poplar wood with superhydrophobic properties and UV resistance was also obtained by Lu et al. [103] using the combined effects of CeO_2_ nanoparticles and octadecyltrichlorosilane (OTS). The wood surfaces were first covered with CeO_2_ nanoparticles and then modified with octadecyltrichlorosilane. The treated wood samples showed excellent performance during 1200 h of accelerated weathering.

Tshabalala and Gangstad [104] coated loblolly pine (*Pinus taeda* L.) wood by the sol-gel process with a combination of methyltrimetoxysilane and hexadecyltrimethoxysilane and evaluated the performance of coated wood surfaces during accelerated weathering. The thin layer of polysiloxane network deposited by the sol-gel process was found to be covalently bounded with the wood surface and exhibited good resistance to photodegradation during accelerated weathering and good resistance to liquid water absorption and to water leaching. The introduction of the UV absorber 2,2,4-trihydroxy-4-[2-hydroxy-3-(3-trimethoxysilylpropoxy) propoxy] benzophenone to the sol-gel system for preparing SiO_2_ wood-inorganic composites has been shown to enhance the photostability of wood against UV light irradiation [105]. However, silane treatment of wood has been shown to have high water repellence but a minor effect on wood sorption behaviour. This influenced the results of research by Donath et al. [106] in which the outdoor weathering of wood treated with silanes caused the checking of wood surface. The combination of UV light stabilizers and silanes showed the increased performance of wood during accelerated weathering. Tshabalala and Sung [107] used methyltrimethoxysilane (MTMOS), hexadecyltrimethoxysilane (HDTMOS) and aluminium isopropoxide (AIP) as precursors for sol-gel deposition of a Al_2_O_3_-SiO_2_ thin film on a loblolly pine (*Pinus taeda* L.) wood surface. It has been shown that this hybrid inorganic-organic thin film deposited on a wood surface was strongly bound to the wood cell wall and protected the wood surface from colour changes caused by UV light. The film deposition was quite resistant to water leaching and to moisture sorption. The same hybrid thin sol-gel films were applied to pine wood (*Pinus radiata* Don.) veneers pretreated with organic UV absorbers and lignin stabilizers, and the weathering performance of such treated veneers was evaluated. It has been found that the combination of light stabilizers and sol-gel thin films deposited on the wood surface can improve the weathering resistance of softwood [108]. Mahltig et al. [109] used silica nanosols modified with an inorganic iron oxide pigment paste for protecting thermally modified Beech (*Fagus sylvatica* L.) wood against accelerated weathering. It has been shown that nanosol-treated wood surfaces are partly protected by the pigment layer during artificial weathering, but the pigment layer also partly eroded in areas without nanosol protection. However, nanosol treatment enhanced the hydrophobicity of the thermally modified wood surface, and the colouration of thermally modified wood can easily be adjusted by the amount of pigment used for nanosol modification. The coating of thermally modified wood surfaces with pigment containing nanosols might be promising for the weathering protection of thermally modified wood.

Zheng et al. [110] reported that the wood samples coated with nanostructural TiO_2_ followed by a mixture of methyltrimethoxysilane and hexadecyltrimethoxysilane exhibited improved resistance to discolouration and weight loss during accelerated weathering. However, after 960 h of exposure to UV radiation and water spray, the specimens showed a drastic decrease in surface water contact angle which can be explained by the effect of the photoactivity of TiO_2_ [111]. Zheng et al. [111] observed that TiO_2_ coating peeled from the wood surface with the rinsing action of the spray water, and the adjacent wood surface degraded because of the photocatalytic activity of TiO_2_. In order to achieve improved weathering resistance by combining TiO_2_ coatings with low surface energy materials, further research is needed to prevent the photocatalytic properties of TiO_2_ or to fix TiO_2_ coatings more firmly in wood [111]. Li et al. [112] fabricated bamboo surfaces with multifunctional properties of hydrophobicity, UV radiation resistance, and fire resistance by coating them with ZnO nanosheets using a hydrothermal method followed by chemical vapor deposition of fluoroalkyl silane. The treated surfaces showed a slight change in colour after 120 h of accelerated weathering and also very good water resistance after 130 h of immersion in water. 

It has been shown that nanoscale films on wood surfaces can be fabricated by the layer-by-layer method, acquiring functional wood surfaces with highly controlled surface chemistry [113]. Rao et al. [114] created a transparent and protective multilayer coating composed of poly(allylamine hydrochloride), poly(styrene sulfonic acid)sodium salt, and nano TiO_2_ films on poplar wood (*Populus ussuriensis* Kom.) surfaces using the layer-by-layer self-assembly technique. The UV stability of wood was enhanced by anatase TiO_2_ in the assembled coating, and the photocatalytic capability of TiO_2_ particles was verified by degrading dyes of rhodamine B and methylene blue. The assembled coating showed superhydrophilicity, but it could be easily altered to hydrophobicity after modification with stearic acid. The layer-by-layer nanoassembly method has been shown to be a simple and practical method for creating thin films with desired layer composition. Lozhechnikova et al. [115] fabricated a protective coating on spruce wood surface via layer-by layer deposition in water using carnauba wax particles and ZnO nanoparticles. This multilayer coating reduced the colour change of the wood surface and made the wood surface superhydrophobic, but further research is required due to the catalytic action of ZnO particles on wax degradation.

### 4.2. Plasma Deposition of Thin Coatings on Wood

Several studies have addressed the weathering-protective properties of coatings deposited on wood surfaces by cold plasma processes. Denes and Young [116] used the cold-plasma approach for deposition and stabilization of protective clear coatings on the Sothern yellow pine wood surface (Denes and Young, 1999). It has been shown that plasma coated polydimethylsiloxane film containing UV absorbers enhanced the resistance of the wood surface to accelerated weathering degradation. Gascón-Garrido et al. [117] used cold plasma spraying at atmospheric pressure for the deposition of copper microparticles on microveneers of Scots pine (*Pinus sylvestris* L.). Plasma-treated wood samples exhibited improved accelerated weathering resistance and reduced blue stain colonisation. The same plasma method was used by Gascón-Garrido et al. [117] for the deposition of thin layer of copper and aluminium microparticles on the surface of Scots pine wood (*Pinus sylvestris* L.). Plasma-treated samples with or without acrylic top coating were exposed to natural weathering for 18 months. It has been shown that the coating of the wood surface with copper reduced the colonisation of staining fungi but did not protect the lignin and hemicelluloses from photodegradation. Plasma treatment with aluminium microparticles has been shown to be ineffective in protecting wood from photodegradation and fungal colonization. Wallenhorst et al. [118] used cold plasma spraying at atmospheric pressure for the coating of beech wood (*Fagus sylvatica* L.) samples with Zn/ZnO particles. They studied the UV-blocking properties of Zn/ZnO coated wood with or without conventional sealer (alkyd or polyurethane commercial coating). It has been shown that Zn/ZnO coatings improved the colour stability of wood with and without additional sealcoating during 50 h of UV exposure without using water spray. However, chemical analysis showed that the application of alkyd sealer to Zn/ZnO-coated samples intensified the photodegradation process which might have been due to the photocatalytic activity of ZnO. The application of Zn/ZnO coating using cold spray plasma at atmospheric pressure followed by a conventional polyurethane sealcoating may be suitable for inline processing and could be a promising, easy way to protect the wood from photodegradation.

## 5. Treatments of Wood with Inorganic Metal Compounds and Bio-Based Water Repellents

### 5.1. Treatments of Wood with Inorganic Metal Compounds

Since lignin is the wood component that is most susceptible to photodegradation, a logical way to increase the resistance of wood surface to UV radiation and weathering would be to use compounds and treatments that can modify lignin. Numerous studies have shown that treatments of wood with aqueous solutions of inorganic metal compounds, such as chromic acid, copper and cobalt chromates, ferric chloride and nitrate, and various manganese, titanium, and zirconium compounds, enhanced the photostability of wood surfaces and improved the durability of clear coatings and stains applied to treated surfaces [119,120,121,122,123]. Many of these treatments change the colour of the wood, which is often a disadvantage in their application in the photostabilization of the wood surface. Chromic acid has been shown to be an effective compound in protecting wood surfaces from weathering [121,122,123,124,125,126], possibly because of the formation of water-insoluble and photostable chromium (III) lignin quinone compounds [127]. It has been shown that a simple dip or brush application of 5% aqueous chromic acid to a wood surface prevented extractive staining, improved dimensional stability, retarded weathering of unfinished wood, and prolonged the life of finishes [121]. During the 1980s in Japan, chromic acid was used as a pretreatment in the finishing of exterior wooden doors with a transparent acrylic urethane coatings [128]. However, due to its toxicity and carcinogenicity, chromic acid is not used on an industrial scale. In addition, chromic acid gives the wood a green/brown colouration. To protect the wood surface from the harmful effects of UV light, less harmful metal treatments that do not cause the unwanted colouring of the wood surface should be used. Thus, for example, manganese compounds that can, to some extent, photostabilize lignin [122] cause the colouration of the treated wood, which also prevents their wider application. Ferric chloride was shown to provide some resistance against natural weathering and restricted colonization by stain fungi [123]. However, Evans and Schmalzl [129] reported that ferric salts were ineffective in reducing weathering probably because no formation of stable, weather resistant lignin–metal complexes occurred [127,130]. Titanates (tetrabutyl, tetraisopropyl, and ethylhexyl titanate) and zirconates (tetrapropyl and tetrabutyl zirconate) are colourless, but have also been shown to be unable to protect lignin from photodegradation during natural weathering [122].

**Figure 3:**
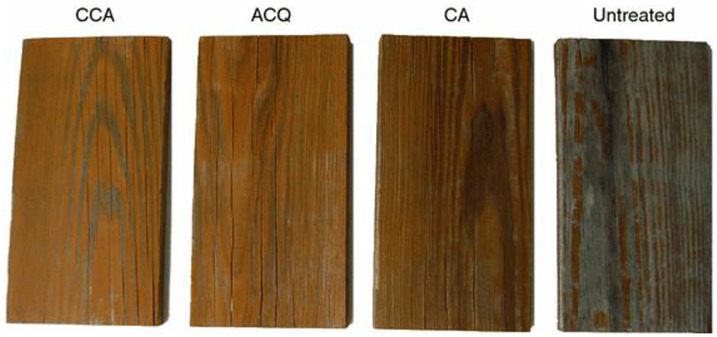
Samples untreated and treated with coper-based preservatives and coated with alkyd-acrylic water-base coating after three years of natural weathering (CCA-chromated copper arsenate, ACQ-alkaline copper quat, CA-copper azole) [136]. Reprinted with permission from Springer Nature: Springer Nature, *Journal of Coatings Technology and Research* (Exterior wood coatings. Part-1: Performance of semitransparent stains on preservative-treated wood, Nejad, M. and Cooper, P.), 2011.

Wood preservatives can also play a role in protecting wood from photodegradation [7]. It was reported that copper-based preservatives, such as chromated copper arsenate and ammoniacal copper quat, reduce the photodegradation of wood by retarding the formation of carbonyl groups and delignification [131,132,133]. The copper ethanolamine treatment was found to be effective in protecting spruce wood (*Picea abies* L. (Karst.)) colour during artificial light exposure [134]. The authors speculated that this might have been due to a reaction of copper ethanolamine with phenolic groups of lignin to form phenolate which retards the formation of phenoxy radicals involved in colour change. Zhang et al. [135] reported that copper monoethanolamine treatment of pine (*Pinus Elliottii*. Engelm.) wood retarded the photodegradation of wood during accelerated weathering. It has also been reported that the performance of semitransparent stains applied to copper-based-preservative-treated wood was improved compared to untreated wood during 3 years of natural weathering [136]. Solvent-based and water-based coatings showed similar water repellent effectiveness on copper-amine-treated wood. The preservative-treated wood samples exhibited less colour change and better visual ratings than untreated wood samples during weathering (Figure 3) [136].

Isaji and Kojima [137] compared the artificial surface weathering of Japanese larch (*Larix kaempferi*) wood pretreated with copper monoethanolamine and chromic acid followed by the application of a semitransparent penetrating stain. They reported that monoethanolamine pretreatment of wood surfaces enhanced the durability of semitransparent penetrating stains after 1000 h of artificial weathering. However, further research is needed to determine whether copper monoethanolamine treatment can be used as an effective photoprotective primer for semitransparent penetrating stains [137]. Chehreh and Mastari Farahani [138] found that treatment of poplar (*Populus deltoids*) sapwood with a nanocopper oxide suspension reduced colour and contact angle changes of a wood surface after 180 days of natural weathering. Vacuum treatment of Southern pine wood with nano-ZnO dispersion has been shown to reduce the greying of wood after 12 months of natural weathering compared to untreated wood samples. Treatments with a nano-ZnO concentration of 2.5% or greater exhibited considerable resistance to water absorption after natural weathering compared to control samples, although moderate checking also occurred. Based on its good properties, nano-ZnO could be one of the components in the future development of multicomponent wood preservatives [40].

### 5.2. Treatments of Wood with Bio-Based Water Repellents

In recent years, there has been an increased interest in using bio-based coatings that are environmentally friendly and consumer friendly. Natural oils and waxes belong to this category of products. Oil treatments enhance the natural wood grain and appearance and reduce surface absorption. It has been reported that linseed and tung oil are very efficient in protecting wood surface against water uptake [139]. Tall oil treatment was not found resistant to accelerated weathering when used alone in Scots pine wood, but in combination with iron oxide, it was found to be effective in reducing the weathering degradation of the wood surface [140]. The natural oils can be modified by introducing new groups to the fatty acid chains to cause the oils to react with the substrate or to increase the compatibility with a potential top coating [70]. The most used method for oil chemical modification is epoxidation, and the most commonly used epoxidized oils are epoxidized soybean and linseed oils (ESO and ELO). These oils have been used successfully alone or in combination with an absorber to formulate the UV system 2-hydroxy-4(2, 3-epoxypropoxy)-benzophenone (HEPBP) [64,141]. Epoxidized soybean oil applied to chemically modified wood with succinic anhydride exhibited efficient wood protection against UV light [142]. Jebrane et al. [143] reported that samples of Scots pine sapwood impregnated with low retentions of epoxidized linseed oil showed similar performance regarding check propagation and moisture uptake as samples with higher retentions after 20 months of natural exposure. ELO samples showed intense discolouration during natural weathering and significant delignification. 

Waxes are important water repellents which can be used for the nonbiocidal protection of wood surfaces in outdoor applications. Waxes increase the water resistance and contribute to the reduction of photochemical degradation [144]. In the past, waxes were used exclusively as additives in water-repellent finishes and preservatives, and today there are commercial wax treatments designed for external use without biocides [145]. Lesar et al. [145] found that that the treatment of wood with high loadings of wax can reduce moisture absorption and slow down the photodegradation process of spruce wood during artificial accelerated weathering. The lowest changes in FTIR spectra and the lowest colour changes showed in samples impregnated with montan wax. To increase the effectiveness of waxes and oils in preventing discolouration due to UV light and water, it is suggested that they should contain photoprotective additives [12] or pigments [146].

## 6. Chemical Modification of Wood

Chemical modification of wood is defined as a chemical reaction between the wood polymeric constituents and a chemical reagent, resulting in the formation of a covalent bond between the reagent and wood substrate [147]. Although most research on chemical modification has been aimed at increasing the dimensional stability and decay resistance of wood [148], there are also studies that have been concerned with the protection of wood against weathering and photodegradation (Table 1).

These studies are included in this chapter. The most common methods of chemical modification are esterification and etherification of hydroxyl groups in the cell wall. The most often used chemicals for wood esterification are anhydrides, acid chlorides, carboxylic acids, and isocyanates, and for wood etherification, alkyl halogenides, epoxides, lactones, and α, β-unsaturated compounds [165].

### 6.1. Acetylation

**Figure 4:**
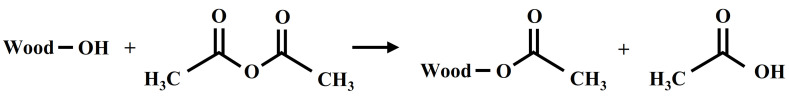
Reaction of wood with acetic anhydride [156]. Reprinted by permission from Springer Nature: Springer Nature, Journal of Journal of Coatings Technology and Re-search (Performance of finishes on wood that ischemically modified by acetylation, Beckers, E.P.J. et al.), 1998.

Among the various wood esterification treatments, acetylation has been the most studied [148]. Acetylation is an example of the chemical modification of wood where the reaction of acetic anhydride with wood results in esterification of the accessible hydroxyl groups in the cell wall (Figure 4), with the formation of byproduct acetic acid [166].

Acetylation is not very effective at protecting wood from photodegradation. Photodegradation of acetylated wood differs from unmodified wood but is not prevented [149,167]. Acetylated wood shows an initial photoprotective effect, and thereafter it begins to fade and grey. It has been reported that acetylated wood has higher checking resistance than unmodified wood when exposed outdoors [150]. Depolymerisation of cellulose and erosion of the middle lamella still takes place after acetylation, but mass loss is reduced and latewood cells maintain their structure [155]. It has been established that acetylation is ineffective in protecting lignin at wood surfaces during accelerated weathering [149,155,168,169], although some photoprotective effects of acetylation to 20% weight gain have been reported on holocellulose and on the morphology of wood cell walls [155,170,171]. However, acetylation is also reported to inhibit discolouration during UV exposure through a reduction of the formation of coloured chromophores on the wood surface [152,153]. Research on extracted lignin acetylated with acetic anhydride showed that acetylation efficiently inhibited the photodiscolouration of lignin under visible and near-UV light irradiation, and this was attributed to the acetylation of phenoxy and aliphatic hydroxyl groups in lignin. Ohkoshi [154] used Fourier transform infrared photoacoustic spectroscopy analysis to characterize the surface changes in acetylated wood during light irradiation and concluded that acetylation restrained the photochemical degradation of wood. Evans et al. [155] reported that wood samples acetylated to low weight gains were less resistant to natural weathering than unmodified samples, but at a higher weight gain of 20%, the rate of degradation (determined by weight loss) was reduced. It seems that the substitution of lignin phenolic hydroxyl groups, which occurs preferentially at low weight gains, increases the photodegradation of wood. The substitution of hydroxyl groups on cellulose in wood, which occurs as a result of acetylation to high weight gains, seems to have beneficial effects on the photostability of cellulose. However, the photoprotective effect of acetylation was found to be lost with prolonged exposure of the acetylated wood to the weather because the deacetylation of the wood surface occurred [155]. Temiz et al. [172] reported lower colour changes of acetylated Scots pine wood (*Pinus sylvestris* L.) compared to unmodified, thermally modified, and silicon modified wood during accelerated weathering. Schaller and Rogez [173] reported that acetylation only partly protects lignin from photodegradation, but there is still a need to protect the acetylated wood with coating that has sufficient UV–VIS light protection with a UV absorber and lignin stabilizer for better long term performance in terms of colour retention. It has also been established that artificial weathering changed the colour of acetylated Scots pine (Accoya) rapidly, after which the surfaces remained stable, as well as lighter and cleaner by visual review [174]. Mitsui [175] reported that the photobleaching of acetylated wood by light irradiation, including UV ray, was caused by mainly visible light without modifying the IR spectra of lignin. Acetylated hornbeam wood (*Carpinus betulus* L.) was reported to be less prone to crack during natural weathering and the accelerating checking test [151], but the modification did not hinder the fading and greying caused by UV light [176].

Acetylation is shown to have a positive effect on the performance of coatings during accelerated weathering [150,156]. It has been shown that the acetylation of wood reduced cracking and flaking of an applied coating when exposed to weathering [156]. This is probably due to the higher dimensional stability of acetylated wood, which reduces the stresses in the coating that originate from the dimensional changes of the substrate. Acetylation in combination with a transparent stain improved the resistance of wood against UV degradation, but the removal of the UV absorber from the stain caused the degradation of the polymers within the coating and the loss of adhesion [156]. Bongers et al. [157] reported that acetylated wood had a significantly better result with respect to long-term coating performance compared to unmodified wood. The acrylic white opaque coating was especially in good condition even after nine years of outdoor exposure. Coating the acetylated hornbeam wood (*Carpinus betulus* L.) samples with boiled linseed oil decreased the rate of colour change and checking [176]. The performance of polyurethane coatings can be significantly improved by chemically modifying rubberwood (*Hevea brasiliensis* (Willd. ex A.Juss.) Müll.Arg.) substrate with benzoyl chloride and acetic anhydride [158].

### 6.2. Chemical Modification with Other Reagents

Evans et al. [159] chemically modified (esterified) thin strips (veneers) of Scots pine wood (*Pinus sylvestris* L.) to high weight gains with benzoyl chloride and assessed the photostability of the chemically modified wood. It has been shown that the esterification of wood with this aromatic acid chloride was effective at photostablising lignin. Benzoylation to high weight gain reduced the quantity of free radicals formed in the wood during exposure to UV light and caused large losses in the tensile strength of thin wood strips. Pandey and Chandrashekar [160] modified Chir pine (*Pinus roxburghii* Sarg) with benzoyl chloride to 19.5 weight gain and analysed colour changes and chemical changes caused by artificial accelerated weathering. They found that the esterification of wood with benzoyl chloride stabilized the wood colour against photodegradation and reduced the lignin degradation and generation of carbonyl groups on the surface of irradiated wood. Pandey and Srinivasa [158] evaluated the weathering performance of uncoated wood modified with benzoyl chloride and benzoylated wood coated with commercially available polyurethane-based transparent and opaque coatings. They found that the esterification of wood with benzoyl chloride slowed down weathering deterioration, but after prolonged exposure, the modified wood exhibited colour darkening/greying. The performance of coatings was significantly improved on a substrate modified with benzoyl chloride after two years of natural weathering.

Prakash et al. [161] modified rubberwood (*H. brasiliensis*) with octanoyl chloride and found no significant effect on the colour of the wood after esterification. The overall colour change (DE*) of modified wood after 250 h of irradiation with simulated sunlight was very small and is probably caused by the esterification of the OH groups of lignin. The modification of rubber wood (*Hevea brasiliensis*) by esterification with palmitoyl chloride has been shown to be effective in photostabilizing wood surfaces to a considerable extent [177]. The dimensional stability of wood was also improved by esterification. Salla et al. [162] modified rubber wood specimens with three fatty acid chlorides (hexnoyl chloride, decanoyl chloride, and tetradecanoyl chloride) and found that the esterification of rubber wood with fatty acid chlorides was effective in restricting photodegradation to some extent, and photoprotection increased with the carbon chain length of fatty acid chloride. However, the authors point out that the range of photoprotection obtained in this work is less than the range of protection obtained by esterification with benzoyl chloride [159,160] and vinyl benzoate [164]. Bhat et al. [163] reported that the esterification of *Acacia mangium* Willd. and *Acacia hybrid* woods with succinic anhydride and propionic anhydride enhanced the weathering resistance of the wood, and succinic anhydride was better in protecting the wood than the propionic anhydride modification. However, the chemical modification of fir wood (*Abies alba* L.) with succinic anhydride did not show a stabilizing effect against irradiation [142]. Jebrane et al. [164] esterified wood with three different aromatic vinyl esters, viniyl benzoate, vinyl cinnamate, and vinyl-4-T-butylbenzoate, and examined the photostability of the modified wood. They found that the modification of wood with vinyl benzoate at high weight gains (>30%) was effective in the protection of lignin and cellulose from photodegradation, while the modification of wood with ninyl-4-T-butylbenzoate did not provide such protection. Vinyl cinnamate has even increased the photodegradation of wood. They concluded that simple, low molecular weight aromatic compounds, such as vinyl benzoate, which by reaction with the molecular constituents of wood can achieve a high degree of substitution of available hydroxyl groups, should be used to protect wood from photodegradation. Vinyl benzoate has no detrimental effects on the tensile strength properties of wood in contrast to benzoyl chloride, which can also modify wood and protect it from photodegradation. Modification with benzoyl chloride has been shown to improve the performance of clear coatings [178]. Chang and Chang [165] used isopropyl glycidyl ether to etherify China fir (*Cunninghamia lanceolata* (Lamb.) Hook.) and maple (*Acer* spp.) wood and evaluate the light stability of modified wood. The etherification of wood by isopropyl glycidyl ether wood was found to inhibit the generation of phenoxyl radicals during UV irradiation and to reduce the colour change of modified woods during UV exposure.

## 7. Modification of Wood and Wood Surface with Thermosetting Resins, Furfuryl Alcohol, and DMDHEU

Melamine-formaldehyde (MF) resin impregnation has shown potential to improve the weathering resistance of wood. Impregnation of pine wood with melamine-formaldehyde or melamine-ammeline-formaldehyde resins was found to enhance the resistance of wood to photodegradation [179]. Rapp and Peek [180] reported that melamine resin protected wood against natural weathering degradation and infestation by wood staining fungi but was not effective in reducing wood cracking. Hansmann et al. [181] observed that the modification of spruce and poplar wood with different melamine formaldehyde resins reduced discolouration and crack formation during long-term artificial weathering. The treatment of wood veneers with a solution containing 30% phenol-formaldehyde (PF) resin and 2% HALS was shown to be as effective as chromic acid at restricting mass and tensile strength losses of veneers during natural weathering [182]. Recent research reveals that impregnation modification with low molecular weight melamine formaldehyde (MF) and PF resins in combination with permanent staining might be an alternative option to surface coatings due to improving weathering protection and additional aesthetic qualities [183,184,185]. Wood modification with thermosetting MF and particularly PF resin was shown to improve the performance of wood coated with acrylic coatings during weathering [184,185]. Recently, Kielmann et al. [183] modified beech wood (*Fagus sylvatica* L.) with a phenolic resin and iron–tannin complexes formulation in order to stain the wood dark and improve weathering performance. The darkest colour and the highest colour stability during weathering were achieved by formulations containing ferric sulphate and tannin. The added additives did not adversely affect the results of wood modification with PF resin. 

The modification of wood with furfuryl alcohol, which can penetrate into wood cells and polymerize in situ during the process, is an environmentally friendly modification process which results in a dark colour, improved dimensional stability, reduced water uptake, and increased resistance to biological degradation [186,187,188]. The furfurylation of wood is a commercialized process, and there are several industrial plants for wood furfurylation in Europe [189]. Temiz et al. [190] reported that the modification of Scots pine (*Pinus silvestris* L.) wood with furfuryl alcohol was ineffective in reducing the discolouration and delignification of wood during accelerated weathering. In comparison with the control deck of ipe wood (.), after three years of outdoor weathering, furfurylated wood decks of radiata pine (*Pinus radiata*), maple (*Acer* spp.), and Southern yellow pine (*Pinus* spp.) showed extensive greying effects, but a smaller total colour change than the control deck, and no signs of black staining (except for Southern yellow pine deck) and no fungal or mould decay [191].

The modification of wood with the *N*-methylol compound 1,3-dimethylol-4,5-dihydroxyethyleneurea (DMDHEU), which is widely used in the textile industry as an antiwrinkling agent, has been shown to be effective in reducing the degradation of cellulose and not very effective in stabilizing lignin during artificial weathering [192]. However, the modification of Scots pine sapwood with modified DMDHEU reduced the discolouration caused by staining fungi, cupping deformation, and surface roughness and waviness compared to unmodified wood panels after 18 months of natural weathering. The performance of acrylic and oil coatings on modified wood was shown to be enhanced compared to unmodified wood [193]. Tomažič [194] also reported improved coating performance on DMDHEU-modified wood after artificial and natural exposure compared to coated unmodified wood. Pfeffer et al. [195] observed the discolouration and lignin degradation of DMDHEU-modified beech and Scots pine wood during 24 months of outdoor exposure. The DMDHEU reduced initial fungal infestation and the speed of liquid water uptake but was not effective in reducing cracking during natural weathering.

## 8. Thermal Modification

Thermal modification is the controlled process of heating the wood at high temperatures between 180 °C and 240 °C under an oxygen free atmosphere, involving either steam, nitrogen or oil [196]. Today, it is today a commercialized technique for increasing the durability and dimensional stability of wood [197]. Thermal modification induces a darker colour of wood which is not stable against light exposure [172,198,199,200,201,202,203]. It was shown that the original dark brown colour of the thermally treated wood spruce and pine wood turned grey during outdoor exposure [204]. However, spruce wood exhibited a lower colour change than unmodified wood after long-term artificial light exposure. Nuopponen et al. [199] reported that the lignin content of thermally modified Scots pine wood samples was higher than the lignin content of unmodified samples after 7 years of natural weathering, which could be due to the increased lignin condensation induced by thermal treatment. Thermal treatment was shown to have no influence on mould and blue stain growth on coated thermally modified spruce and pine wood during natural weathering, although the moisture content of modified wood was found to be lower compared to unmodified wood [205]. Miklečić et al. [206] reported that the thermal modification of beech wood, ash wood, and hornbeam wood retarded discolouration compared to unmodified wood but induced similar chemical changes as in unmodified wood during UV light exposure. Cracking due to dimensional changes was higher in thermally modified wood, and oil finish reduced cracking. Thermal modification was found to be ineffective in improving the UV resistance of *Larix* spp. wood over long-term UV radiation [202]. It has also been reported that thermally modified spruce and pine wood showed the same level of cracking as unmodified wood during natural exposure, which was not prevented by applications of low-build stains or oils. It can be concluded that thermally modified wood has poor resistance against weathering, and surface treatment with coatings is required for both protection and aesthetic reasons. A more detailed review of the recent results in the field of weathering performance of thermally modified wood is given in the paper prepared by Jirouš-Rajković and Miklečić [207].

## 9. Conclusions

The summary of the protective functions against weathering and the disadvantages of the investigated methods of wood protection is presented in Table 2.

Research into methods to protect wood surfaces from weathering and UV radiation has been conducted for many years and is still an attractive area of research. The effectiveness of protection of most methods has been determined by accelerated exposure; however, in order to gain a complete insight into the protected properties of each method, it is necessary to conduct natural exposures over a longer period. Many methods of weathering protection are still in the phase of academic research and far from commercial application, and the search for effective, nontoxic, inexpensive, and environmentally friendly methods for protecting wood from weathering continues. Applying protective coatings still seems to be the simplest method of protecting wood from weathering for commercial applications. Some modification methods have been shown to be ineffective in improving the weathering resistance of wood but effective in extending the durability of coatings on modified wood, thus shortening coating maintenance intervals and repair costs. A possible solution to increase the weathering resistance of wood is to combine different protection methods. There is a growing emphasis on the use of nanomaterials in the protection of wood surfaces, which entails questions about the dangers of these materials to human health and the environment. Currently, no method provides complete protection from weathering because weathering is caused by numerous factors. The emphasis of most methods is on protecting the wood surface from UV radiation and water absorption which are the most important factors of weathering.

## Figures and Tables

**Table 1:** Findings on the protection of chemical reagents against wood weathering.

Chemical Reagent	Findings	Reference
acetic anhydride	- does not prevent photodegradation - increases resistance to checking - inhibits discolouration during UV exposure - restrains photodegradation of wood - reduces degradation at higher weight gain of 20% - has positive effect on performance of coatings during weathering	[149,150,151] [150,151] [152,153] [154] [155] [150,156,157,158]
benzoyl chloride	- increases performance of coatings during natural weathering - reduces lignin degradation	[158] [159,160]
palmitoyl chloride	- contributes to photostabilization	[161]
fatty acid chlorides	- reduces photodegradation	[162]
succinic anhydride propionic anhydride	- enhances weathering resistance	[163]
vinyl benzoate	- protects cellulose and lignin from photodegradation at high weight gains	[164]
vinyl cinnamate	- increases photodegradation of wood	[164]
vinyl-4-T-butylbenzoate	- does not protect cellulose and lignin from photodegradation	[164]
isopropyl glycidyl ether	- reduces colour change during UV exposure	[165]

**Table 2:** Protective functions of method of surface protection against weathering and their disadvantages.

Methods	Protective Function against Weathering	Disadvantage
Treatments with photostabilizers	absorption of energy from UV radiation prevention of photodegradation of wood and coating	migration of organic photostabilizers toxicity of inorganic photostabilizers
Finishing with coatings	protection of wood surface from solar radiation and moisture (depending on the formulation)	pigmented coatings hide the attractive appearance of wood transparent coatings transmit harmful UV radiation
Thin film deposition onto wood surfaces	protection of wood surface from photodegradation, bacteria, and fungi	too expensive for wide commercial application [161]
Treatments with inorganic metal compounds	increasing photostability and durability of clear coatings	changing wood colour toxicity [162]
Treatments with bio-based water repellents	environmentally friendly reducing water uptake	need regular and rapid renewal
Chemical modification	increasing dimensional stability and decay resistance reducing photodegradation (except vinyl cinnamate and vinyl-4-T-butylbenzoate) increasing performance of coatings	toxicity vinyl cinnamate increases photodegradation
Modification with thermosetting resins, furfuryl alcohol and DMDHEU	increasing dimensional stability reducing water uptake increasing resistance to biological degradation	changing wood colour toxicity
Thermal modification	reducing water uptake	resulting dark colour is not stable against light exposure [165]

## Data Availability

The data presented in this study are available upon request from the corresponding author.
